# Dyschromatopsia: a comprehensive analysis of mechanisms and cutting-edge treatments for color vision deficiency

**DOI:** 10.3389/fnins.2024.1265630

**Published:** 2024-01-17

**Authors:** Zihao Yang, Lin Yan, Wenliang Zhang, Jia Qi, Wenjing An, Kai Yao

**Affiliations:** ^1^Institute of Visual Neuroscience and Stem Cell Engineering, Wuhan University of Science and Technology, Wuhan, China; ^2^College of Life Sciences and Health, Wuhan University of Science and Technology, Wuhan, China

**Keywords:** color blindness, achromatopsia, cone, mechanism, treatment

## Abstract

Color blindness is a retinal disease that mainly manifests as a color vision disorder, characterized by achromatopsia, red-green color blindness, and blue-yellow color blindness. With the development of technology and progress in theory, extensive research has been conducted on the genetic basis of color blindness, and various approaches have been explored for its treatment. This article aims to provide a comprehensive review of recent advances in understanding the pathological mechanism, clinical symptoms, and treatment options for color blindness. Additionally, we discuss the various treatment approaches that have been developed to address color blindness, including gene therapy, pharmacological interventions, and visual aids. Furthermore, we highlight the promising results from clinical trials of these treatments, as well as the ongoing challenges that must be addressed to achieve effective and long-lasting therapeutic outcomes. Overall, this review provides valuable insights into the current state of research on color blindness, with the intention of informing further investigation and development of effective treatments for this disease.

## 1 Introduction

**Figure 1:**
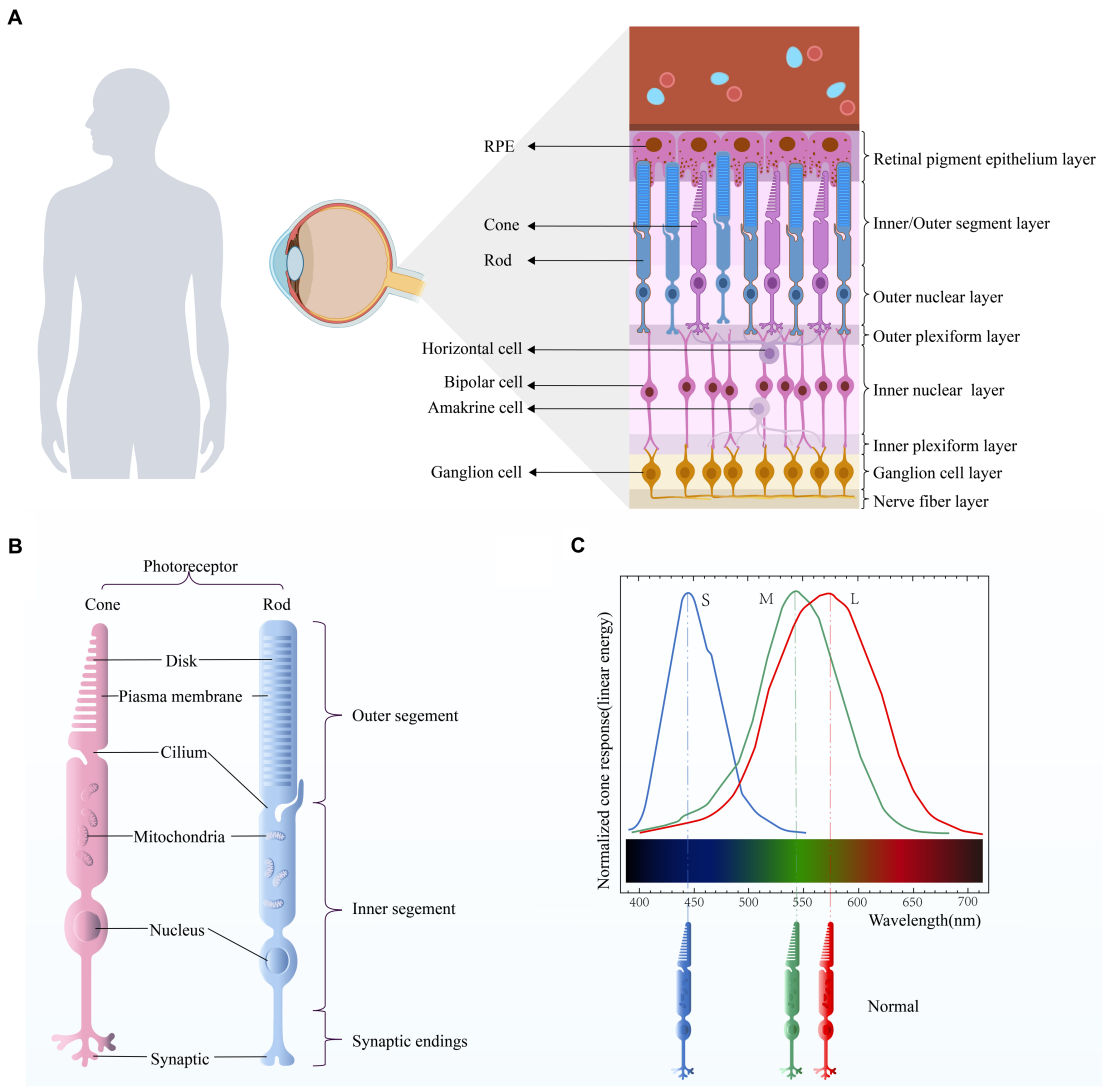
**(A)** Retinal stratification. The retina is mainly composed of retinal pigment epithelial (RPE) cells, cone cells, rod cells, horizontal cells, bipolar cells, amacrine cells, and ganglion cells. **(B)** Photoreceptor structure. There are two types of photoreceptors in the human retina, namely the cone and the rod. Their structures are similar and can be roughly divided into three parts: outer segment, inner segment, and synaptic ending. **(C)** Optic cone cell absorption spectra. Different types of cone cells have different peak sensitivities.

Vision is one of the most important sources of information for human beings. The human visual system relies on two types of photoreceptors located in the retina (Figure 1A), namely rods and cones. The human retina contains around 90 to 120 million rod cells, while only 4 to 6 million cone cells. Rod cells are located in all areas except the central fovea, while cone cells are clustered in the central fovea. Among them, the rod cells are responsible for night vision, that is, black and white vision. They are highly sensitive and can reliably receive the signal of a single photon. However, this high sensitivity makes rods susceptible to saturation; thus, for example, human rod monochromats (persons whose retina lacks functional cones) are light-blinded even at low diurnal levels of illumination. The cones are responsible for daytime vision and color vision. And the sensitivity of cones is 100 to 1,000 times less than that of rods. The main cause of the differing sensitivity of rods and cones is the ability of cones to terminate their photo response faster. This ensures that the cone does not saturate and provides useful vision at the maximum intensity available in nature (Astakhova et al., 2015). When cone function is impaired, it can result in color blindness.

Color blindness, a type of cone dysfunction syndrome (CDS), is a color vision disease that includes both congenital and acquired forms. Patients with this disease are unable to distinguish one or more colors in the natural spectrum. Congenital color vision disease is an inherited retinal disease (IRD) that is passed down through chromosomes, affecting both males and females but showing a greater prevalence in men. Acquired color vision diseases have various causes, including ocular and neurological disorders, some metabolic disorders, drug toxicity, and exposure to certain solvents (Swanson and Cohen, 2003). Furthermore, research indicates that prolonged exposure to alcohol during adolescence and young adulthood can result in visual deficits and impair color vision (Brasil et al., 2015).

The origins of our understanding of color blindness can be traced to a 1,602 account by a Chinese medical scientist. Coincidentally, British physicist and chemist Robert Boyle also described a case of “visual impairment” in an article in 1688. Boyle wrote that the maid sometimes “wanted to gather violets,” but “she knelt where the violets grew, and could not distinguish weeds and flowers by color, only by shape or feeling” (Simunovic, 2010). About 100 years later, the British chemist and physicist Dalton also discovered color blindness as a result of his own and his brother’s deficiencies and published “On Color Blindness” in 1793 (Emery, 1988). In honor of his achievements, congenital red-green color blindness is also known as Daltonism.

With the development of modern scientific understanding, including genetics, there has been an improved understanding of color blindness. Congenital color blindness is divided into Achromatopsia (ACHM), red-green colorblindness, and blue-yellow colorblindness.

Achromatopsia is an autosomal recessive genetic disorder of the retina. This disease affects three types of cone cells in the retina, resulting in complete cone cell dysfunction, poor vision, complete loss of color vision, leaving only the ability to differentiate between light and dark, often accompanied by photophobia, nystagmus, and other phenomena.

Red-green color blindness (anerythrochloropsia), a recessive disease carried on the X chromosome, is divided into protanopia (red blindness) and deuteranopia (green blindness). This condition is the most prevalent type of color vision disorder, constituting around 95% of all color vision disorders (Salih et al., 2021) and is the most common single locus genetic disorder (Mancuso et al., 2009). Patients are unable to distinguish between red and green.

Blue-yellow color blindness (tritanopia), an autosomal dominant disorder, is characterized by the inability to recognize blue and yellow, although the patient can recognize the red and green spectrum (Weitz et al., 1992a).

In addition to these diseases with severely impaired color vision, CDS also encompasses some diseases of lesser severity, such as color weakness, oligocone trichromacy (OT) and bradyopsia (Aboshiha et al., 2016). The boundary between color blindness and color weakness is not very clear, and some patients are close to colorblindness when the light is dim. Color weakness includes panchromatic weakness, red-green color weakness and blue-yellow color weakness.

Patients with panchromatic weakness display a reduced level of color vision impairment in contrast to those with achromatopsia, without any associated abnormal vision or complications. While the ability to perceive colors under bright lighting conditions is preserved, distinguishing between them becomes challenging in low-light settings.

The red-green color weakness (dyserythrochloropsia), including the red color weakness (protanomalia) and the green color weakness (deuteranomalia), affects the patient’s ability to distinguish red and green. In dim lighting, the patient is close to red-green color blindness, while in bright lighting, the patient is close to normal. The prevalence rate of protanomalia is approximately 1.08%, while the prevalence rate of deuteranomalia is approximately 4.63% (Salih et al., 2020).

The blue color weakness (tritanomalia), similar to the red-green color weakness, and the ability to distinguish blue is poor. The patient is close to blue-yellow color blindness when the light is dark, and close to normal when the light is bright. The prevalence rate is about 0.2% (Salih et al., 2020).

Currently, the rate of color blindness is approximately 5% for male sand 0.7% for females. The frequency of color blindness gene carriers is estimated to be 8.98%. Among them, the prevalence of total color blindness is estimated to be 1 in 30,000 (Kohl and Hamel, 2013), the prevalence of red blindness is approximately 1.01%, the prevalence of green blindness is approximately 1.27%, and the prevalence of blue-yellow blindness is approximately 0.2% (Salih et al., 2020).

OT is characterized by severe impairment of cone function on ERG assessment coupled with normal or near-normal color discrimination. It was first described in 1973 by Van Lith, who reported a boy that, despite his poor vision and reduced photopic ERG responses, had nearly normal color vision. This was hypothesized to be due to a low number of normal functioning cones (from the Greek oligos for ‘few’), which retained their normal distribution proportions between the three cone types, hence preserving trichromatic vision.

Bradyopsia is a rare condition that affects vision. The term “bradyopsia” is from the Greek words for slow vision. In affected individuals, the eyes adapt more slowly than usual to changing light conditions. Bradyopsia was first reported in 1991 in four Dutch patients, who demonstrated an abnormally long interval of suppression in their ERG amplitude responses to the second of a pair of bright stimuli flashes. This was postulated to be due to a deficit in the normally fast regeneration of the visual pathway signaling processes. The term bradyopsia (Greek for slow vision) was devised in 2004 to describe this stationary retinal phenotype, wherein affected patients had difficulty in adapting to sudden changes in cone-mediated luminance levels and difficulty in seeing moving objects (Aboshiha et al., 2016).

It has been suggested that people with color vision disorders have relevant advantages in nighttime vision tasks (Chapanis, 1947). Yet this assumption clearly does not stand up to close scrutiny (Simunovic et al., 2001). Color vision disorders not only affects the patient in daily life, such as the inability to recognize traffic lights and an inability to see the colorful world, but also has a negative impact on the patient’s employment, making them unable to engage in aviation, navigation, engineering, and military fields of work (Holroyd and Hall, 1997). Therefore, it is necessary to diagnose color vision disorders as early as possible (Hathibelagal, 2022).

In order to rescue the vision of patients with color vision disorder, it is an urgent task to solve the pathological mechanism of the color vision disorder and find a correct and effective treatment. This paper briefly summarizes the progress in related fields.

## 2 Clinical symptoms and pathological mechanism

### 2.1 Total color blindness (achromatopsia)

#### 2.1.1 Clinical symptoms of achromatopsia

Patients with achromatopsia have complete loss of cone function, loss of color vision and poor vision (<0.1), accompanied by nystagmus, photophobia (Simunovic and Moore, 1998) and eccentric fixation (Sundaram et al., 2014). Hyperopia is common (Wissinger et al., 2001; Kelly et al., 2003) and there are extensive reports of refractive errors (Haegerstrom-Portnoy et al., 1996). However, the rod cells of patients with achromatopsia have normal function and a faster dark adaptation ability than in normal subjects. Fundoscopy examination is normal in most cases, but there are exceptions (Goodman et al., 1963; Khan et al., 2007), and findings of fundus pigmentation, small macular granularity, and macular defects have also been reported (Khan et al., 2007).

As genetic technology continues to advance, our comprehension of achromatopsia deepens. Furthermore, newly emerging technologies and equipment offer enhanced support for researching, diagnosing, and treating ACHM, including the implementation of optical coherence tomography (OCT) and adaptive optics scanning laser ophthalmoscopy (AOSLO). OCT has the capability to assess the retinal structure *in vivo* through the outer hyperreflective bands that correspond to various aspects of photoreceptor cells. Despite this, traditional OCT devices lack the lateral resolution necessary to differentiate individual photoreceptors (specifically, the structure of rods and cones). Therefore, OCT only provides a general assessment of photoreceptor structure in ACHM. However, with AOSLO, we can capture non-invasive images of rods and cones with cellular level resolution.

As a result of advancements in technology and equipment, additional symptoms linked to pathogenic mutations have been uncovered in addition to the typical ones. Certain studies propose that GNAT2 mutations can permit some ACHM patients to retain portions of their color vision (Michaelides et al., 2003; Rosenberg et al., 2004). A study suggested that the retention of color vision may be related to variants that result in some functional protein products (Rosenberg et al., 2004). Although ACHM is traditionally deemed a disease that remains stable throughout one’s lifetime, recent research indicates that patients with CNGA3 (Thomas et al., 2012), CNGB3 (Thiadens et al., 2010; Thomas et al., 2012), and PDE6C (Thiadens et al., 2010; Georgiou et al., 2019) variants have evidence of progressive cone cell involvement, that even affect rod cells. Subsequent studies have also demonstrated that, in addition to ACHM, mutations in PDE6C can cause cone dystrophy (CD) and cone-rod dystrophy (CRD; Thiadens et al., 2009; Grau et al., 2011; Weisschuh et al., 2018). Full field ERG showed severe cone system dysfunction, but shortwave sensitivity was relatively preserved, similar to another rare form of ACHM (GNAT2-ACHM; Michaelides et al., 2003) and similar to blue cone monochromatic lesions (Gardner et al., 2014). Myopia and slowly progressive maculopathy are common features among patients with PDE6C mutations (Georgiou et al., 2019). In ACHM patients caused by CNGA3 or CNGB3 mutations, the ellipsoid zone (EZ) band (also known as the inner segment/outer segment [IS/OS] band) was disrupted or deleted in approximately 70% of cases (Sundaram et al., 2014; Langlo et al., 2016). Furthermore, foveal hypoplasia with minimal foveal pit formation has been observed in all subjects with ATF6-ACHM to date (Kohl et al., 2015; Xu et al., 2015). And relevant research data show that ACHM caused by ATF6 gene mutation has almost no vertebral structure and fewer targets for cone gene therapy (Mastey et al., 2019).

#### 2.1.2 Classification of achromatopsia

**Figure 2:**
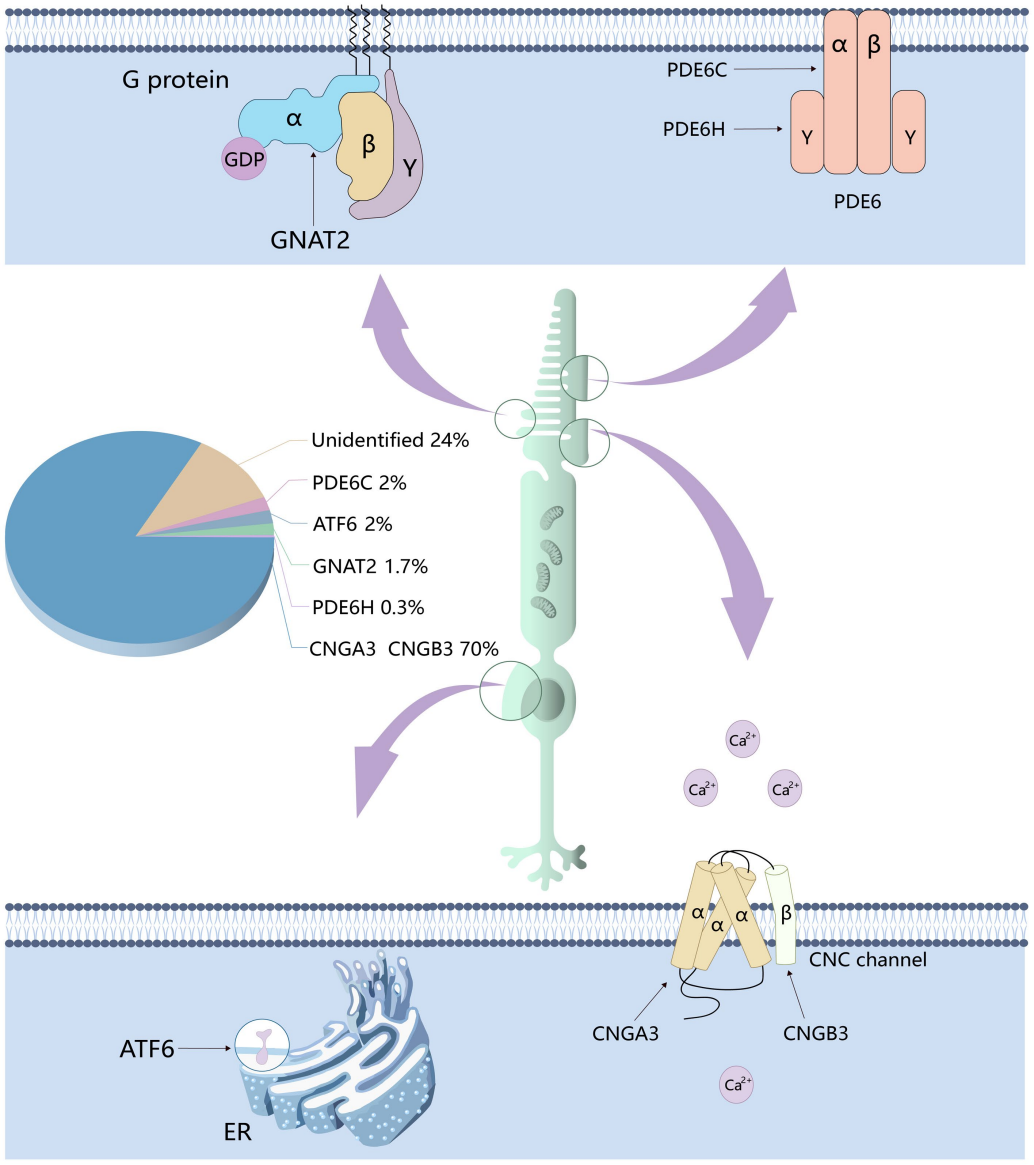
The genes responsible for achromatopsia and the proportion of cases thereof. Six genes related to achromatopsia have been identified: CNGA3, CNGB3, PDE6C, PDE6H, GNAT2, and ATF6. CNGA3 and CNGB3 encode the α and β subunits of CNG channel respectively, which affect the flow of calcium ions. PDE6C and PDE6H encode the α and γ subunits of PDE respectively, which affect the concentration of cGMP. GNAT2 encodes cone cell specificity G protein α subunit, which affects the activity of PDE.

Currently, six genes that cause achromatopsia have been discovered, including CNGA3, CNGB3, GNAT2, PDE6C, PDE6H and ATF6 (Felden et al., 2019). Among them, CNGA3, CNGB3, GNAT2, PDE6C and PDE6H all encode cone cell-specific proteins. Over 70% of ACHM cases are caused by mutations in CNGA3 and CNGB3 genes with the proportion of ACHM cases caused by CNGB3 mutation reached 50% (Kohl et al., 2005; Mayer et al., 2017; Georgiou et al., 2020; Brunetti-Pierri et al., 2021). Mutations in the other four genes account for less than 6%–8% of cases (Felden et al., 2019; Georgiou et al., 2021). The remaining 24% of unexplained cases may be due to undetected pathogenic genes or unidentified mutations (Weisschuh et al., 2018, 2020a; Brunetti-Pierri et al., 2021; Figure 2).

#### 2.1.3 Pathological mechanism of achromatopsia

Rod cells and cone cells consist of four primary structural and functional regions: outer segment, inner segment, cell body and synaptic terminal (Figure 1B). The outer segment is densely packed with a dense stack of membrane disks that are spaced at intervals of approximately 28 nm intervals. These disks contain the visual pigments (rhodopsin in rod cells and cone pigment in cone cells) and other transduction components, either as transmembrane or peripheral membrane proteins. Visual pigment is the most abundant protein in the outer segment. An intriguing variation between rods and cones is that the rod disks (excluding nascent disks at the base of the outer segment) are entirely internalized and thus physically separated from the plasma membrane, whereas the cone disks remain as the folding of the plasma membrane. The open cone-shaped discs provide a greater surface area, enabling speedy transfer of substances between the exterior and interior of cells. This facilitates the efficient transfer of chromophores for pigment regeneration and swift calcium dynamics during light adjustment (Fu and Yau, 2007).

Rod cells and cone cells undergo continuous renewal of their outer segments (Young, 1967; Young and Droz, 1968). The newly formed disk at the base of the outer segment gradually pushes the previously formed disk toward the top. The intersegmental disk, situated at the apex of the lateral segment, sheds daily and is phagocytized by adjacent retinal pigment epithelial cells (RPE; Young and Bok, 1969). The rate of disc formation and shedding is roughly equivalent, thereby ensuring a relatively constant outer segment length in the adult retina.

The inner segment contains a substantial amount of endoplasmic reticulum (ER) and Golgi apparatus. To satisfy the elevated metabolic energy requirements related to phototransduction, a significant number of mitochondria are present. All proteins that travel to the outer segment must traverse the narrow channel between the outer segment and the inner segment.

The synaptic end converts light signals into electrical signals and transmits them to subsequent neurons in the retina: bipolar cells and ganglion cells. In the dark, a stable inward current (“dark current”) is generated by the influx of cations on the outer segment membrane, which depolarizes the rod or cone and maintains the stable release of glutamate from the synapse. The light signal stops the influx of cations (“photosensitive” conductance, consisting of cGMP-gated channels) to stop the dark current and produce membrane hyperpolarization. This hyperpolarization reduces or terminates the release of glutamate. The signal is then further processed by other neurons in the retina before being transmitted to the higher center of the brain.

Human vision of sunlight and color depends on three types of retinal cone photoreceptors with complete functions. However, cone cells are not only responsible for providing color vision, they are also responsible for mediating high acuity spatial vision, achromatic black-white vision (Neitz and Neitz, 2017) and central visual acuity (Kohl et al., 2002). Cone cells sensitive to short wavelengths (blue), medium wavelength (green), and long wavelength (red), namely S-cones, M-cones and L-cones, express visual pigments (cone proteins) that are sensitive to specific wavelength of light, respectively, with peak spectral sensitivity of 419, 531, and 560 nm (Dartnall et al., 1983; Figure 1C). But different classes of cones respond to a wide range of wavelengths of light, so they have overlapping sensitivity curves (Simunovic, 2010). The cone pigment is composed of 11-cis retinal and opsin. All eutherian mammalian cone pigments share the same 11-cis retinal chromophore (Wald, 1968). Binding of opsins to chromophores red-shifts the absorption spectrum of the chromophore, and differences in the amino acid sequence between opsins are responsible for the different spectral characteristics of each cone pigment (Wald, 1968; Kosower, 1988; Chen et al., 1989). The cone cells respond very quickly to changes in light. Even in dim light (when the response is slowest), only 20 ms is needed to reach the peak response of the cone to the superimposed flash (van Hateren and Lamb, 2006). As the background intensity increases, the response of the cones becomes faster, and in very bright light, the photopic vision system can detect flickers of more than 100 Hz in the surrounding retina (Tyler and Hamer, 1990). In the human retina, L-cones and M-cones constitute approximately 95% of the total cone population. They are densely packed in a hexagonal pattern in the central fovea, the foveola. The S-cone is located in the periphery of the retina and is not present in the fovea in humans (Curcio et al., 1991; Mustafi et al., 2009). Color recognition depends on the differential excitation of cone pigments by light stimulation of specific wavelengths and the correct processing of receptors after stimulation. Color vision impairment occurs when the function of one or more cone photoreceptors is lost or altered (caused by mutations, deletions, or rearrangements of opsin genes).

Total color blindness is a rare autosomal recessive retinal disease caused by the complete loss of the function of the three cone pigments, also referred to as rod monochromatism. In addition to ATF6 (activating transcription factor 6), CNGA3, CNGB3, GNAT2, PDE6C, and PDE6H genes all encode the phototransduction cascade components of cone cells, which are responsible for converting light signals into electrical and calcium signals (Michalakis et al., 2022). Mutation of any one of these genes will result in the paralysis of the entire phototransduction cascade system.

The CNGA3 (ACHM2, OMIM600053) gene is located on chromosome 2 and encodes the alpha subunit of CNG channel (Cyclic nucleotide gated ion channel) in cones (Table 1). The CNGB3 (ACHM3, OMIM605080) gene is located on chromosome 8 and encodes the beta subunit of the CNG channel (Michalakis et al., 2017) in cones. CNGB3 is the major ACHM pathogenic gene in Europe and North America (Weisschuh et al., 2020b). CNG channels are heterotetramers composed of alpha subunit and beta subunit. They are the key component of the cone cells phototransduction cascade assembly. Although recent studies have shown that ACHM due to CNGA3 or CNGB3 mutations cannot be clinically distinguished, they are quite different (Kohl et al., 2002). Previous studies have shown that CNGA3 is an ion conduction subunit and the CNGA3 homologous channel is fully functional in heterologous expression systems (Gerstner et al., 2000; Faillace et al., 2004). However, when the CNGB3 is expressed alone, it cannot form functional homologous channels in the heterologous expression system (Chen et al., 1993; Gerstner et al., 2000). Therefore, the beta subunit is only considered to confer specific biophysical properties of the CNG channel complex and to regulate the function of the cone CNG channel, while the alpha subunit is considered to be the main subunit (Kaupp and Seifert, 2002; Schön et al., 2013). Related researches also point out that most mutations in CNGA3 are missense mutations (Kohl et al., 1993; Biel and Michalakis, 2009; Sun and Zhang, 2019), causes the CNG channel to lose its function. The working mechanism of CNG channels is that the high concentration of cGMP outside the photoreceptor keeps the CNG channel open to cation influx in the dark, while light stimulation leads to a decrease in cGMP level, and subsequently the CNG channel closes. This closure terminates the stable inward current, thereby generating a membrane hyperpolarizing signal that reduces glutamate release from photoreceptor synapses (Müller and Kaupp, 1998). Some studies have pointed out that the degeneration of cone cells caused by spontaneous CNG channel activity may be the pathogenic factor of color blindness (Zheng et al., 2022).

**Table 1:** ACHM pathogenic genes.

Gene	Full name	Location	Gene size (KB)	Exons	CDS length (bp)	Gene type	Gene ID
CNGA3	Cyclic nucleotide gated channel alpha 3	2q11.2	53	9	2,085	Protein coding	1,261
CNGB3	Cyclic nucleotide gated channel beta 3	8q21.3	170	19	2,430	Protein coding	54,714
GNAT2	G protein subunit alpha transducin 2	1p13.3	16.6	9	1,065	Protein coding	2,780
PDE6C	Phosphodiesterase 6C	10q23.33	69	22	2,577	Protein coding	5,146
PDE6H	Phosphodiesterase 6H	12p12.3	9.6	4	252	Protein coding	5,149
ATF6	Activating Transcription Factor 6	1q23.3	197.8	17	2,013	Protein coding	22,926

**Figure 3:**
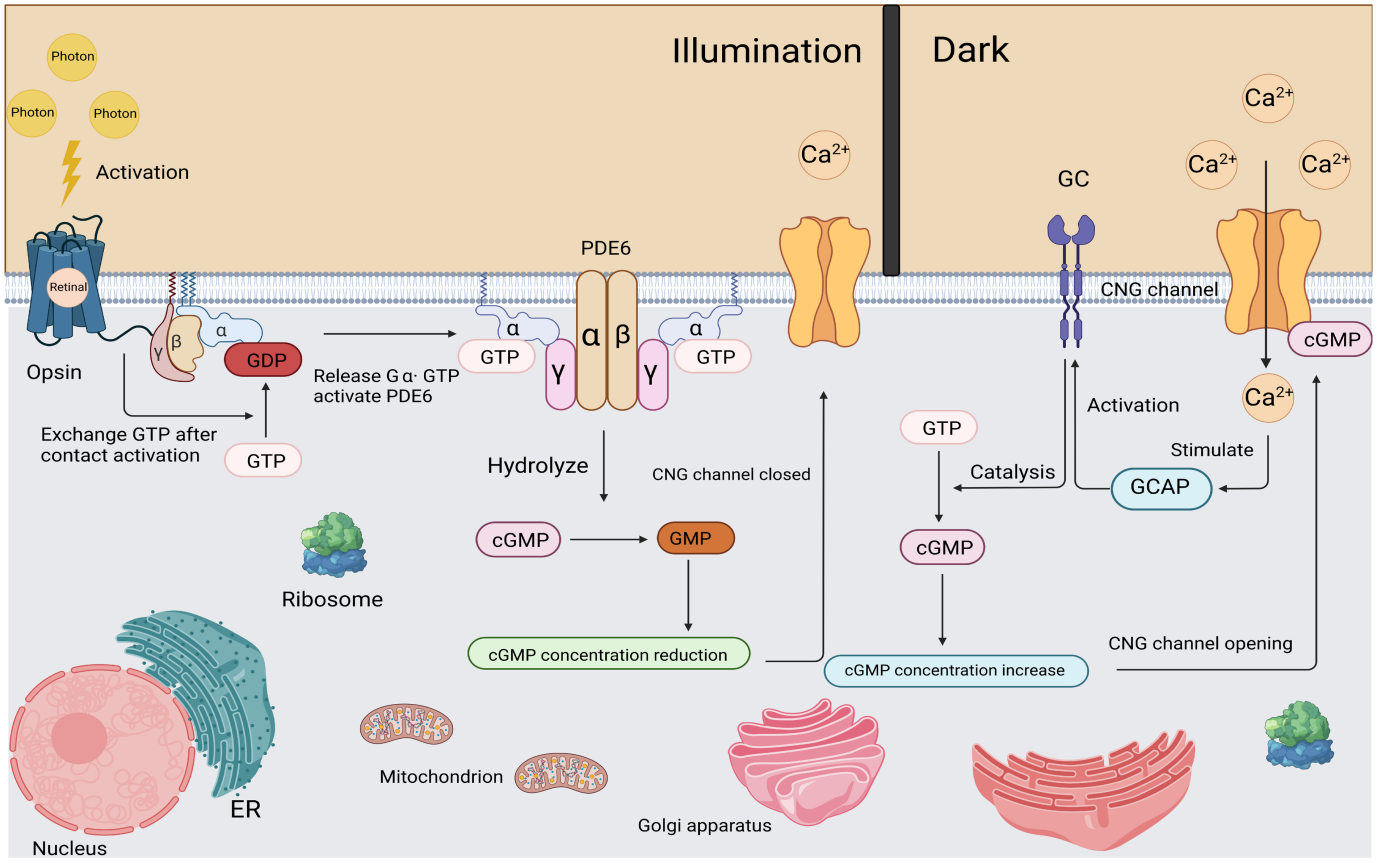
Light activation and recovery of cone cells. After corresponding stimulation, Gα subunit was released from Gβγ subunit and the exchange between GDP and GTP was completed. The subsequently activated Gα subunit binds to the γ subunit of PDE and causes it to contract to complete the activation of PDE. Activated PDE begins to hydrolyze cGMP efficiently. As the cGMP concentration decreases, the CNG channel closed. In a dark environment, a certain concentration of cGMP maintains the opening of CNG channels.

The GNAT2 (ACHM4, OMIM139340) gene is located on chromosome 1 and encodes cone cell specificity alpha subunit, a heterotrimeric G protein coupled to cone cytochrome in the outer segment of the cone photoreceptor. The beta and gamma subunits interact with photoactivated photopigments to exchange GTP with GDP, thereby releasing the α-subunit. The activated GTP • transduction complex binds and activates phosphodiesterase, which subsequently hydrolyzes cGMP, thereby effectively reducing its intracellular concentration. This leads to the closure of cGMP-gated channels, followed by membrane hyperpolarization (Stryer, 1991). All GNAT2 mutations identified to date result in premature translation termination and protein truncation at the carboxyl terminus (Aligianis et al., 2002; Kohl et al., 2002). A recent study by Felden et al. pointed out that the prevalence of ACHM caused by GNAT2 mutations was estimated to be 1.7% in a cohort of 1,116 independent ACHM families (Felden et al., 2019; Figure 3).

PDE6C (ACHM5, OMIM600827) is located on chromosome 10 and encodes the catalytic alpha subunit of pyramidal phosphodiesterase (PDE; Uzunov and Weiss, 1972). PDE is the third component of the vertebrate phototransduction system, and is a tetrameric protein consisting of two equally active catalytic subunits α and β and two identical inhibitory subunits γ (Baehr et al., 1979; Hurley and Stryer, 1982). In the dark, two γ subunits act as inhibition subunits by binding to two catalytic subunits and preventing the hydrolysis of cGMP. PDE is anchored to the disk membrane by isoprenylation of the C termini of the two catalytic subunits (Li et al., 1990; Catty et al., 1992; Qin et al., 1992). It was reported that PDE* hydrolyzes cGMP at a rate close to the limit set by aqueous diffusion, with a Km of ~10 μM and a K_cat_ of 2,200 s^−1^ (Leskov et al., 2000). After the initiation of phototransduction, transducing proteins shrink their inhibitory γ subunit activates PDE6C. Followed by the last step of the cascade, the hydrolysis of cGMP by active PDE6C leads to the closure of the extrapyramidal cation channel. Then hyperpolarization of the photoreceptor membrane was performed (Zhang and Cote, 2005). The PDE6C gene mutation leads to its loss of function, which reduces the hydrolysis level of cGMP in cells, thereby causing the cGMP level to rise. The increase of cGMP level mainly leads to the excessive opening of cGMP-gated channels in the extrapyramidal segment, resulting in an unrestricted inflow of Ca^2+^. CGMP gated channel belongs to the family of cyclic nucleotide gated (CNG) channels and is a non-selective cationic channel (Kaupp and Seifert, 2002). This channel is located on the plasma membrane of photoreceptor cells and is the last component in the activation stage of phototransduction. In the dark, the basal concentration of one to several micromoles of cGMP keeps a small number of CNG channels open (Yau, 1994). The drop in cGMP concentration upon exposure to light causes the channel to close rapidly with a sub-millisecond delay (Karpen et al., 1988). Related studies have shown that uncontrolled increased concentrations of cytoplasmic cGMP and Ca^2+^ ions can lead to the death of cones (Iribarne and Masai, 2017). A recent study by Weisscheh et al. estimated that the prevalence of PDE6C mutations was 2.4% in a cohort of 1,074 independent ACHM families (Weisschuh et al., 2018). Studies of ACHM caused by PDE6C mutations have shown conflicting results, and Thiadens et al. concluded in a cohort of 5 PDE6C patients (20%) that ACHM is usually a progressive disease (Thiadens et al., 2010). However, Hirji et al. concluded in a cohort of one PDE6C subject (2%) that ACHM is mainly a quiescent disease (Hirji et al., 2018). This issue requires further research.

PDE6H (ACHM6, OMIM601190) is located on chromosome 12 and encodes the pyramidal cell phosphodiesterase inhibitory γ subunit (Kohl et al., 2012). The PDE6H mutation functionally means that PDE activity is continuously increased, cGMP levels in the outer cone segment are decreased, and cGMP gated channels are permanently closed, which is similar to permanent light stimulation (Lisman and Fain, 1995). One study estimated a prevalence of 0.3% in 680 independent ACHM cohorts (Kohl et al., 2012).

ATF6 (ACHM7, OMIM605537) is a widely expressed gene located on chromosome 1 that encodes cyclic AMP-dependent activating transcription factor-6 alpha that plays a key role in unfolded protein response (UPR) and endoplasmic reticulum homeostasis (Walter and Ron, 2011). ATF6 is located at the autosomal recessive cone-rod dystrophy (CORD8) locus, which was previously mapped to the 11.53 cM region on chromosomes 1q12-q24 in a Pakistani family (Khaliq et al., 2000; Ismail et al., 2006). As one of the three transmembrane proteins that regulate the UPR, ATF6 is activated upon ER stress, transcriptionally upregulates ER chaperones and ER protein folding enzymes, helping to alleviate ER stress and restore cellular homeostasis (Walter and Ron, 2011; Chan et al., 2016; Wang and Kaufman, 2016). When cones trapped in ER stress due to excessive light damage, ATF6 mediates early UPR to relieve ER stress and restore homeostasis (Nakanishi et al., 2013). Additionally, ATF6 also activates IRE1 (Inositol requiring enzyme-1), which mediates the late UPR, during which the activation of ATF6 decreases, and severe or sustained ER stress can lead to apoptosis (Lin et al., 2007; Nakanishi et al., 2013). Apoptotic cell death caused by ER stress has been recognized as a common pathway in retinal degenerative diseases, including ACHM (Thapa et al., 2012). Pathogenic sequence variants in the ATF6 gene cause dysfunction of this critical signaling pathway. As one of the most active cells in human metabolism, this is particularly harmful to photoreceptor cells (Sung and Chuang, 2010; Wong-Riley, 2010). A recent study showed that the ATF6 pathway is essential for color vision not only within photoreceptors, but also in the inner layer of the retina and is a potential target for the treatment of inherited or acquired retinal diseases (Ansar et al., 2015). Research shows that the proportion of ACHM cases caused by ATF6 mutation is less than 2%.

Due to the scarcity of cone cells and the fragility of COS (cone outer segment), as well as a number of other reasons, the phototransduction mechanism of cone cells cannot be accurately described (Fu and Yau, 2007). However, its mechanism is generally similar to the phototransduction mechanism of rod cells, and there is nothing inherently different between the processes of activation of the cone and rod phototransduction (Astakhova et al., 2015). Here, we take the phototransduction mechanism of rod cells as an example:

First, activation of the Rhodopsin, a photon of light is absorbed by the rhodopsin molecules in the outer segments of the rods, isomerizing the 11-cis retinal (the chromophore of cone and rod cells) to all-trans retinal, thereby inducing a conformational change in the structure of the rhodopsin molecule, becoming the activated form R*.

Second, activation of the G-protein. All proteins move within (or on the surface) of the disc membrane, so they diffuse laterally; thus, activated R* and inactivated G contact and bind instantaneously. In this R*-G state, the GDP molecule bound to the G protein alpha subunit (Gα) is released, allowing the GTP molecule in the cytoplasm to bind to its place. This process, called nucleotide exchange, activates the G-protein (forming G-GTP), which then dissociates from the R*. Most importantly, the activated rhodopsin (R*) is not altered in any way by this interaction, so it can come into contact with another G protein molecule by diffusion, triggering nucleotide exchange again. While R* remains active, this process can be repeated indefinitely, so R* can be viewed as an enzyme that catalyzes the activation of numerous molecules of G proteins. Activated G protein is denoted as G*.

Third, activation of the PDE. As a result of lateral diffusion, G* contacts the PDE and binds to one of its two regulated γ subunits, thereby partially activating the PDE in the form of PDE* that we denote. Subsequently, the second G* can bind to the second γ-subunit to fully activate the PDE. Unlike step 2, this activation does not have an amplification effect: a single G* can only activate at most one PDE catalytic subunit.

Fourth, hydrolysis of Cyclic GMP (cGMP). In the dark resting state, there is a stable balance between cyclic GMP synthesis by guanylate cyclase (GC) and the slow hydrolysis of cyclic GMP by normal PDE, so there is a stable, moderate and appropriate cytoplasmic concentration of cyclic GMP (a few micromoles). Upon exposure to light stimulation, disc proteins are activated (Steps 1–3), and the resulting PDE* activation increases the rate of cyclic GMP hydrolysis, thereby decreasing cyclic GMP concentrations.

Finally, closure of Ion Channels. At its resting concentration in the dark, cyclic GMP binds to, and holds open, a proportion of the ion channels in the cell’s plasma membrane. Although this proportion is quite small (typically just a few percent), the number of open channels is sufficient to conduct the substantial cation current (tens of pA) that flows into the outer segment in darkness. When the concentration of cyclic GMP drops in the light, cyclic GMP unbinds from the channels, causing the channels to close, and thereby generating the cell’s electrical response—a reduction in the circulating current and a consequent hyperpolarization (Lamb and Pugh, 2006; Figure 3).

While the underlying mechanisms exhibit a broad similarity, notable distinctions exist. Firstly, in cones, it is the cone visual pigment rather than rhodopsin that receive incoming light signals. Secondly, *in vitro* biochemical analyses have consistently demonstrated that the activation rate of cone visual pigment is 2–5 times slower compared to rhodopsin. It has been confirmed in humans (Vissers et al., 1998), mice (Kojima et al., 2014) and chickens (Imai et al., 1997; Kojima et al., 2014). Thirdly, the fast quenching of cone R* is ensured by phosphorylation via cone-specific rhodopsin-kinase (G protein coupled receptor kinase 7, GRK7), whose level of expression and specific activity are substantially higher than those of GRK1 in rods (Rinner et al., 2005; Tachibanaki et al., 2005; Wada et al., 2006; Arinobu et al., 2010; Vogalis et al., 2011). Fourth, rod cells can respond to a single photon, whereas cone cells need to be stimulated by more than a hundred photons. Fifth, in the rod outer segment (ROS), the disc membrane is separated from and surrounded by the plasma membrane. Instead, the cone outer segment (COS) is formed by a stacked invagination of the plasma membrane (Mustafi et al., 2009). As a result, the rod discs are isolated from the extracellular space by the plasma membrane, while the cone discs are open to the extracellular matrix. The structure of the outer segment of the cone greatly increases the surface area of its disc membrane and the surface-to-volume ratio of the cone. The continuous and open structure of the outer segments of the cones facilitates the rapid response of light transmission and metabolism in the cones, and ensures the rapid transport of substances between the cones and the interphotoreceptor matrix (IPM; Yau, 1994). Sixth, the chromophore quenching and regeneration rate of cone cells are faster than rod cells (Wang and Kefalov, 2011).

To put it succinctly, the process of light transduction in cone cells can be described as follows: Upon exposure to light, the cone visual pigment molecules within the cells are stimulated. This stimulation activates the transducing protein α subunit (GNAT2 = G_α_). At the guanosine binding site, guanosine diphosphate (GDP) is exchanged for guanosine triphosphate (GTP), resulting in the release of the inhibitory βγ subunit and the formation of the activated form of the G protein (G _α_-GTP). Activated Gα-GTP leads to a significant increase in phosphodiesterase (PDE) activity. PDE is responsible for hydrolyzing cyclic guanosine monophosphate (cGMP) and effectively reducing its concentration in the outer segment of the cone cell. The decrease in cGMP concentration causes the closure of heterotetrameric cGMP-gated cation channels (CNGA3/CNGB3), resulting in membrane hyperpolarization of the cone cell. The membrane hyperpolarization leads to a decreased release of the excitatory neurotransmitter, glutamate. Finally, the cone photoreceptor cells recover from the light response through a series of quenching/terminating reactions of the activated phototransduction proteins, which ultimately restore the cells to a dark-adapted state. These intricate steps ensure efficient light detection and signal processing in cone photoreceptor cells.

### 2.2 Red-green color blindness

#### 2.2.1 Clinical symptoms of red-green color blindness

The genes responsible for coding the red cone protein and green cone protein are located on the X chromosome at Xq28, encode the protein components of the light-sensitive photopigments that are specifically expressed in the L cones and M cones (Neitz and Neitz, 2021), with mutations leading to red-green color blindness. Patients with red-green color blindness commonly experience have symptoms such as decreased vision, photophobia, nystagmus and myopia (Gardner et al., 2009). Despite female carriers being asymptomatic, confocal AOSLO has demonstrated variably reduced cone density, increased spacing, and disrupted organization, with phenotypic variability likely relating to random X-chromosome inactivation (Carroll et al., 2010).

#### 2.2.2 Pathological mechanism of red-green color blindness

**Figure 4:**
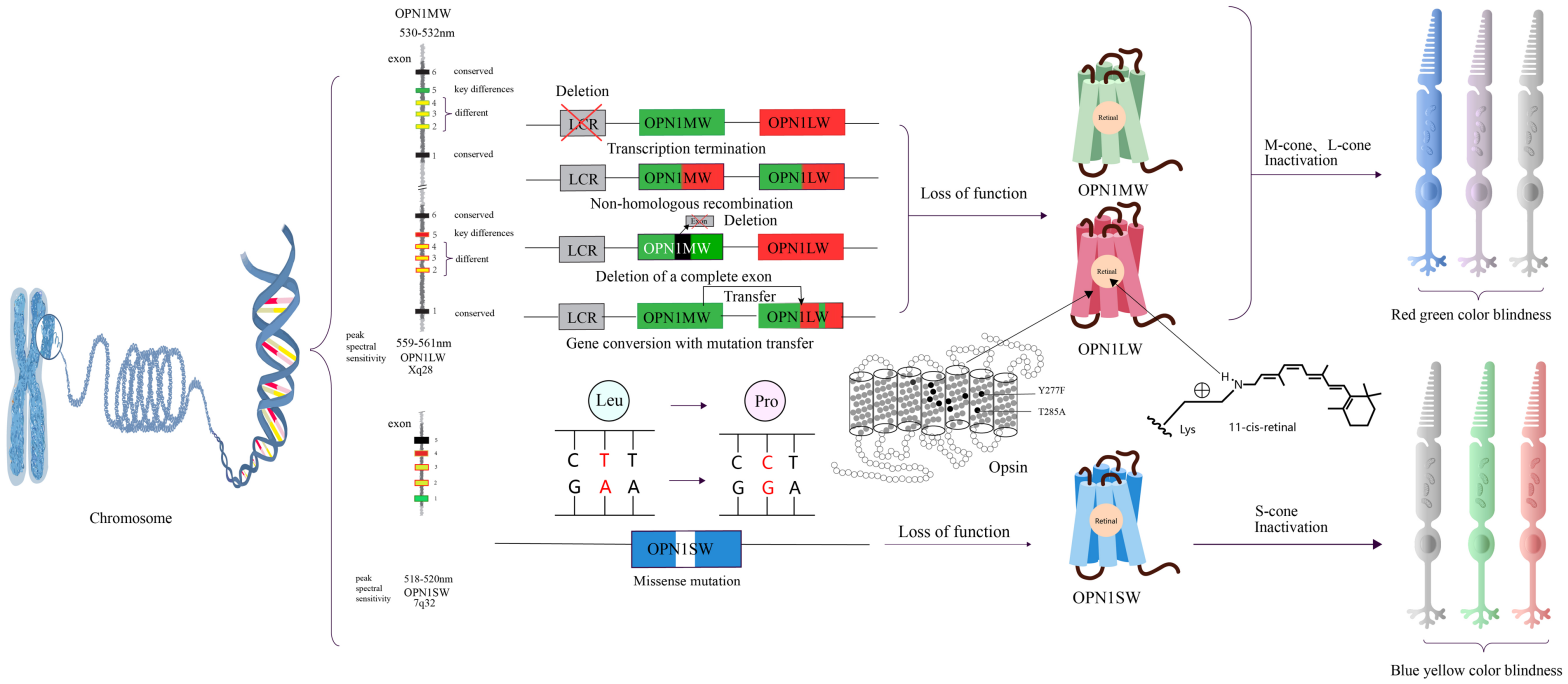
The pathogenic mechanisms of red-green color blindness and blue-yellow color blindness and opsin structures. There are four mutation patterns for OPNIMW and OPNILW, resulting in the loss of function of the corresponding visual pigment and causing red-green color blindness. For OPNI S W, there is only one missense mutation pattern, leading to blue-yellow color blindness. Y277F and T285A are amino acids that determine key differences between retinoids. The gray cone represents loss of function.

The red-green color blindness is an X-linked recessive genetic disorder caused by mutations in two genes, OPN1MW (Opsin 1 medium wavelength gene, OMIM300821) and OPN1LW (Opsin 1 long wavelength gene, OMIM300822), resulting in a lack of long-wavelength photosensitive opsin protein and medium-wavelength photosensitive opsin protein (Table 2). In humans, OPN1MW and OPN1LW genes are linked together on the X chromosome in a head-to-tail arrangement (Vollrath et al., 1988). Both OPN1LW and OPN1MW genes have 6 exons each (Nathans et al., 1986). OPN1LW and OPN1MW are almost identical to each other, with more than 98% nucleotide sequence homology. Because of their similarity, L and M opsin genes are prone to unequal homologous recombination, which has profound implications for visual function (Neitz and Neitz, 2011). Exons 1 and 6 are highly conserved with almost no changes (Winderickx et al., 1993). Exon 5 encodes amino acid differences that functionally separates the L and M photopigments (Neitz et al., 1991). Exons 2, 3 and 4 differ between L-opsin (red) and M-opsin genes (green). The selective expression of OPN1LW and OPN1MW is regulated by specific proximal promoters and a single upstream locus control region (LCR), ensuring that only one opsin gene is expressed in each cone photoreceptor (Wang et al., 1992; Yamaguchi et al., 1997; Smallwood et al., 2002). At present, there are four pathological mechanisms of red-green color blindness: the first is the partial or complete deletion of the locus control region, resulting in transcriptional termination of an opsin gene array (Nathans et al., 1989). The second is non-homologous recombination between long-wave opsin and medium-wave opsin gene arrays followed by inactivating mutations. The third is the deletion of a complete exon in the single opsin gene array (Ladekjaer-Mikkelsen et al., 1996). The fourth is the gene conversion with mutation transfer between OPN1LW and OPN1MW (Reyniers et al., 1995). In addition, in some cases, it is also associated with cone dystrophy (Gardner et al., 2010; McClements et al., 2013; Figure 4).

**Table 2:** Opsin genes.

Gene	Full name	Gene type	Gene ID	Location	Exons
OPN1SW	Opsin 1, short wave sensitive	Protein-coding	611	7q32.1	5
OPN1MW	Opsin1, medium wave sensitive	Protein-coding	2,652	Xq28	6
OPN1LW	Opsin 1, long wave sensitive	Protein-coding	5,956	Xq28	6

### 2.3 Blue-yellow color blindness

#### 2.3.1 Clinical symptoms of blue-yellow color blindness

The gene responsible for encoding the blue cone protein is located on chromosome 7 at position 7q32. This gene, referred to as OPN1SW (Opsin 1 short wavelength gene, OMIM190900), has a nucleotide sequence similarity of only about 40% with the other two opsin genes. Mutations in OPN1SW lead to abnormal functionality of the blue cone protein, resulting in a condition known as blue-yellow color blindness. Affected individuals experience difficulty in effectively perceiving colors in the short wavelength range, but they can still distinguish colors in the medium and long wavelength regions.

#### 2.3.2 Pathological mechanism of blue-yellow color blindness

Blue-yellow color blindness is a dominant genetic disorder with a simple pathology. Missense mutations in the gene encoding the blue cone opsin protein result in amino acid substitutions within the blue cone opsin sequence. As a consequence, individuals with these mutations experience blue-yellow color blindness (Weitz et al., 1992a, b). Other studies have suggest that blue-yellow color blindness could be linked to defects in OPN1SW splicing defects and haploinsufficiency (Neitz et al., 2020; Figure 4).

## 3 Treatment method

### 3.1 Treatment of ACHM

Currently, there are no pharmaceutical treatments or therapeutic methods exist for achromatopsia (Simunovic, 2010). The management of this condition primarily involves specialized genetic counseling to provide guidance and support to affected individuals and their families. Low vision devices may also be used as visual aids to assist with visual tasks. To alleviate symptoms of photophobia, individuals with achromatopsia may also benefit from wearing colored contact lenses or glasses (Kohl and Hamel, 2013). A study has reported that the use of red glasses or red contact lenses may provide relief for patients experiencing photophobia (Park and Sunness, 2004). Clinical trials have shown that these visual aids such as colored contact lenses or glasses are not fully effective in addressing the challenges related to color vision disorders (Martínez-Domingo et al., 2019).

**Figure 5:**
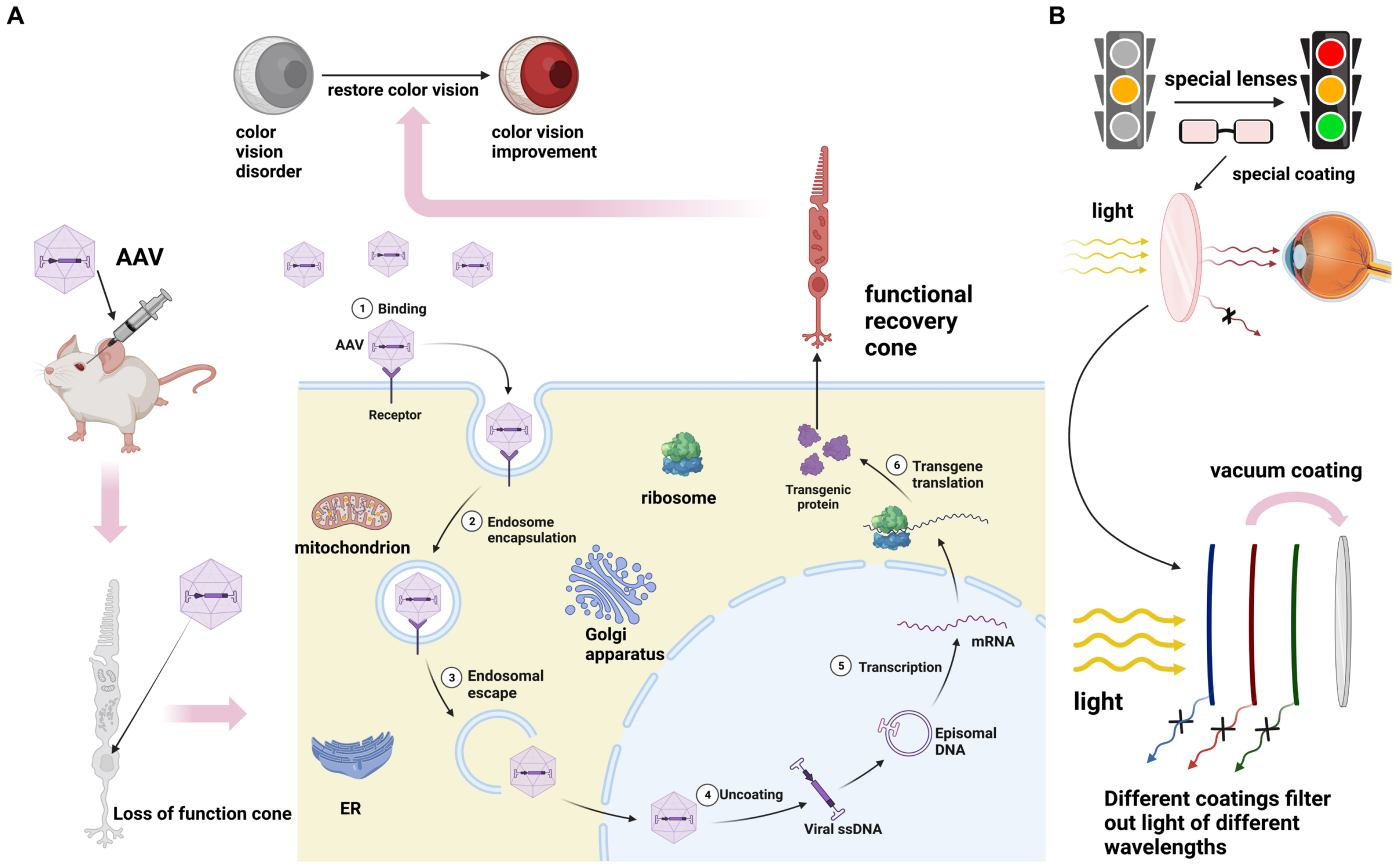
**(A)** The principle of gene therapy for ACHM. After intraocular injection of AAV, the exogenous normal genes carried by AAV replace the mutated genes to perform their functions. Transcription translation produces proteins with normal functions to save color vision. **(B)** The principles of physical therapy for red-green color blindness and blue-yellow color blindness involve applying special coatings on lenses to filter out specific wavelengths of light. It’s important to note that currently, all treatment methods for these three types of color blindness are still immature.

However, it is noteworthy that the coding sequences of all genes associated with total color blindness are relatively small, typically less than 2,600 base pairs (BP). This compact size allows for their encapsulation within adeno-associated virus (AAV) gene therapy vectors, which holds promise for potential gene therapy interventions (Kotterman and Schaffer, 2014). Although AAV has inherent limitations as a gene delivery system, advancements in vector engineering offer the potential to develop improved recombinant AAV (rAAV) variants with enhanced delivery capabilities. These engineered rAAV vectors aim to overcome the size constraints and improve the efficiency of gene transfer, thereby expanding their therapeutic potential in various gene therapy applications (Michalakis et al., 2022). Currently, several studies have provided compelling evidence demonstrating the efficacy of gene-based or alternative therapies in restoring pyramidal function in animal models of achromatopsia (ACHM) with various genotypes (Alexander et al., 2007; Komáromy et al., 2010; Michalakis et al., 2010; Carvalho et al., 2011; Figure 5). Studies have reported that in cases of achromatopsia (ACHM) caused by GNAT2 mutations, cone cells exhibit a relatively well-preserved state. This finding suggests a potential broad therapeutic window for interventions targeted specifically at cone cells (Georgiou et al., 2020). In a study conducted on Gnat^cpfl3^ (Cone photoreceptor function loss-3) mice, the delivery of GNAT2 cDNA was shown to result in the restoration of cone function, as evidenced by improvements observed in photo-adapted electroretinogram (ERG) and optomotor response (OMR) tests (Alexander et al., 2007). Another study used AAV5 vector expressing human CNGB3 to treat two color blind dog models and successfully improved their response to maze tasks and functional vision (Komáromy et al., 2010). Among mammals, only primates have trichromatic vision. Therefore, these studies based on non-primate color blindness models still need to be further developed. And it is worth noting that the cone residues of ACHM caused by different pathogenic gene mutations are different, which cannot be generalized.

Interestingly, previous research has produced conflicting results. Specifically, several studies have demonstrated that the therapeutic window for gene therapy decreases with age (Komáromy et al., 2010; Carvalho et al., 2011). However, studies have indicated that a clear relationship between the degradation of retinal structure or function in individuals with ACHM and age has not yet been established. This implies that gene therapy may have a wider potential window for intervention in specific cases than previously assumed (Sundaram et al., 2014).

It is important to emphasize that in current research, there is a shift toward utilizing subretinal (SR) injection as a delivery method instead of intravitreal injection. This change is driven by the limitations of intravitreal injections in effectively targeting the outer retina and the increased risk of missing the desired location in the inner retina (MacLaren et al., 2016). Although gratifying progress has been made in gene therapy, safety studies on sheep and non-human primate models have shown that injection can lead to inflammation. However, it is reassuring to note that several gene therapies have demonstrated a generally acceptable safety profile, indicating the feasibility of their clinical application in the future (Seitz et al., 2017; Ofri et al., 2018; Reichel et al., 2018; Tobias et al., 2019).

Another research study has reported therapeutic effects of CNTF (ciliary neurotrophic factor) on both rod and cone photoreceptors in degenerative diseases. This finding highlights the potential of CNTF as a treatment option for preserving and promoting the function of photoreceptor cells affected by degenerative conditions (Lipinski et al., 2015). And it has been proved that CNTF has a neuroprotective effect on rod photoreceptor cells in several animal models (Wen et al., 2012). Recent studies have shown that CNTF can also produce protective effects on cone photoreceptors (Li et al., 2010; Talcott et al., 2011), and promote regeneration of the outer segments of degenerated cones (Li et al., 2010). These research findings have garnered significant attention from the scientific community. Currently, a phase I/II clinical trial (National Clinical Trial number: NCT01648452) is underway to evaluate the efficacy of CNTF treatment in patients with CNGB3 mutations. However, the trial results have indicated that CNTF did not lead to a substantial improvement in cone function. This suggests that there may be species-specific differences in the response of cone CNGB3 to CNTF, which highlights the need for further investigation and understanding of the underlying mechanisms in humans (Zein et al., 2014). Interestingly, the combined administration of CNTF has been shown to overcome a major limitation of gene therapy alone, which is the inability to achieve therapeutic effects in the majority of dogs over 1 year old (Komáromy et al., 2013). This promising finding highlights the potential of CNTF as an adjunct therapy in combination with gene therapy, suggesting a new avenue for therapeutic intervention in these cases.

ACHM caused by CNGA3 and CNGB3 mutations accounts for 70% of all cases, making them the most extensively studied genes. In fact, relevant progress has been made in North America, Europe and the Middle East, and the first phase of clinical trials for the treatment of color blindness with AAV carriers has been started, which are Germany (CNGA3, NCT02610582), the United States and Israel (CNGA3, NCT02935517), the United States (CNGB3, NCT0259922) and the United Kingdom (CNGB3, NCT03001310; Table 3). Several clinical trials have already yielded results:

**Table 3:** AAV treatment project.

NCT ID	Gene	Vector	Capsid	Participating country	Current status	Phase
NCT02610582	CNGA3	rAAV.hCNGA3	rAAV8	Germany	Completed recruiting	I/II
NCT02935517	CNGA3	rAAV2tYF-PR1.7-hCNGA3 (‘AGTC-402’)	AAV2tYF	United States and Israel	Recruiting	I/II
NCT02599922	CNGB3	rAAV2tYF-PR1.7-hCNGB3	AAV2tYF	United States	Recruiting	I/II
NCT03001310	CNGB3	AAV2/8-hCARp.hCNGB3	AAV5	United Kingdom	Completed recruiting	I/II
NCT03758404	CNGA3	AAV2/8-hG1.7p.coCNGA3	AAV5	United States and United Kingdom	Completed recruiting	I/II
NCT03278873	CNGB3	AAV2/8-hCARp.hCNGB3	AAV5	United States and United Kingdom	Completed recruiting	LTFU

Following successful gene augmentation therapy in a naturally occurring ovine model (Banin et al., 2015; Gootwine et al., 2017), a phase I/II a human trial was initiated in some research centers in the United States (ClinicalTrials.gov, ID NCT02935517). In this trial, patients are treated with a single subretinal injection of an AAV2tYF capsid variant carrying the CNGA3 transgene under control of the engineered PR1.7 cone-specific opsin promoter in their worse eye. Although there is some improvement in the patient’s vision after 1 year of treatment, there is no evidence of improvement in color vision (McKyton et al., 2021).

In another study (ClinicalTrials.gov, ID NCT02610582), researchers obtained even more exciting results. This gene therapy study for achromatopsia found an excellent safety profile associated with subretinally delivered AAV8.CNGA3 and functional improvement in patients 1 year after treatment, although the absence of randomized concurrent control individuals precludes determining a cause-and-effect relationship. Improvement in functional variables, such as visual acuity, contrast sensitivity, and color vision, were noted despite the limited cohort size. In addition, patient-reported outcome measures provided additional support for a possible beneficial treatment effect. For example, the ability to differentiate colors improved after treatment with AAV8.CNGA3, as did measures for vision and identification of letters and numbers—key tasks in everyday life. As such, we found that targeting cone photoreceptors for gene supplementation with recombinant AAV8 can be applied safely and successfully. Of more importance, these data provide evidence that cone photoreceptor function can be gained in adult patients with complete achromatopsia (Fischer et al., 2020).

In a study to treat CNGB3-associated ACHM (ClinicalTrials.gov, ID NCT03001310), researchers achieved promising results. This is a phase 1/2, open-label, nonrandomized, dose-escalation/expansion study. In this trial, researchers treated 23 participants (11 adults, 12 children) with AAV8-hCARp.hCNGB3. It resulted in an adverse event (AE) profile that was expected and manageable. Overall, AAV8-hCARp.hCNGB3 demonstrated an acceptable safety and tolerability profile. There was no systematic pattern of change from baseline to week 24 for any specific efficacy assessment between the treated and untreated eye across the entire study population, within either cohort (adult or child), or with respect to dose. However, favorable changes were observed for individual participants across several assessments, participants’ performance improved after treatment on several efficacy assessments including PA (light sensitivity) and VRQoL (vision-related quality of life). Improvements in several efficacy parameters indicate that AAV8-hCARp.hCNGB3 gene therapy may provide benefit (Michaelides et al., 2023). The research results of other projects have not been announced yet.

Due to the low prevalence of ACHM caused by GNAT2 mutations, a limited number of studies have focused on phenotypic analysis despite advances in the field of gene therapy (Georgiou et al., 2020). The proportion of ACHM cases caused by PDE6C, PDE6H, and ATF6 mutations is relatively low, and therefore, the research focus on these genes is not as extensive as that of CNGA3 and CNGB3. Currently, there are no ongoing clinical trials specifically targeting these genes.

### 3.2 Treatment of red-green color blindness

To date, there is still no effective treatment for red-green color blindness. Treatment approaches currently being explored can be broadly categorized into physical therapy and gene therapy.

In the realm of physical therapy, significant efforts are being directed toward the research and development of wearable devices. At present, there are special lenses that are covered with a variety of coatings to filter out specific wavelengths of light (Randhawa et al., 2010). These innovative lenses aim to enhance color perception and improve the overall visual experience for individuals with red-green color blindness. In addition, a kind of dyed contact lenses has been developed to improve patients’ color perception, which has the advantages of non-toxicity, low cost and customizability (Badawy et al., 2018). Another study showed that contact lenses made of gold nanoparticles (GNPs) and hydrogel matrix also have the function of improving color vision (Salih et al., 2021). At present, the leader in the color blindness glasses market is Enchroma, which first released its products in 2012. The glasses use a multi-notch filter to remove the overlap of red and green. The lens material used in this product is Trivex, which is lighter, thinner, and stronger than commonly used glass materials (CR-39 and polycarbonate), by coating the lens surface with a dye with a narrow absorption band to avoid affecting the patient’s normal ability to distinguish colors. However, one study tested its products using color assessment and diagnosis (CAD), and the results showed that the products did not significantly improve the symptoms of patients with color vision disorders. In addition to Enchroma, there are VINO, Colorlite and other companies engaged in the production of related products. Nevertheless, the current corrective eyeglasses available for red-green color blindness present several limitations. They tend to be expensive, bulky, and incompatible with other vision correction lenses. Furthermore, their effectiveness and stability in improving color perception are often unsatisfactory.

In the field of gene therapy, there is currently no mature and reliable drug or treatment available for red-green color blindness. However, certain research findings have shown promising indications for researchers in this field. For instance, a study demonstrated that adult primates with red-green color blindness, when supplemented with a third opsin, were able to achieve trichromatic vision. This suggests that trichromatic vision can be attained by introducing a single additional type of cone, without the need for early developmental processes. These findings offer a promising outlook for gene therapy in treating color vision disorders in adult humans (Mancuso et al., 2009). However, it should be noted that other studies have indicated that none of the conducted studies so far have definitively shown that the experimental animals have gained the ability to perceive new colors. Additionally, it should be noted that there is currently no evidence indicating that these experimental animals can differentiate between the colors red and green, despite their ability to detect both colors against a gray background (Cornelissen and Brenner, 2015). The findings of this study raise doubts regarding the efficacy of gene therapy in treating red-green color blindness, suggesting that it is premature to conclude that gene therapy can effectively address this condition in humans.

However, there is also encouraging news. Reports indicate that gene therapy has shown potential in rescuing the remaining M-cones in aged OPN1MW^−/−^ mice (M-opsin knockout), offering hope for the treatment of color vision deficiency (Deng et al., 2019). In addition, a study provided encouraging evidence by demonstrating that the introduction of exogenous human opsin resulted in the regeneration of the outer cone segment and restoration of M cone function in OPN1MW^−/−^ mice (Deng et al., 2018). The findings from these studies hold promise for the potential development of a cure for individuals with color vision disorders, particularly middle-aged and elderly patients.

Another study showed that the electroretinogram (ERG) function of M-cone was successfully restored in congenital S-cone monochromatic rat model by using a vector containing rat M-cone-opsin (Zhang et al., 2011). This result provides a direction for human treatment of color blindness. Recent studies have shown that there are still residual L-cone and M-cone at 1.5 mm in the center of the retina of red-green color blindness patients (Luo et al., 2015). This retinal structural evidence suggests that red-green color blindness deserves consideration for human clinical trials of L-cone and M-cone gene therapy (Cideciyan et al., 2013).

In addition to these scientific studies, certain technology companies, such as Google and Apple, have also taken initiatives to address the challenges faced by individuals with red-green color blindness. For instance, Google is developing an interface and application specifically designed to mitigate the negative impact of color vision impairment on individuals with red-green color blindness. Apple is committed to manufacturing end products for patients with red-green color blindness, improving the impact of red-green color blindness on patients’ daily life from a mechanical level.

### 3.3 Treatment of blue-yellow color blindness

There is no effective treatment for blue-yellow blindness. Clinical outcomes of assistive devices like glasses or contact lenses have generally been underwhelming. The relatively low prevalence of blue-yellow color blindness and its limited impact on patients, as S-cones only make up around 5% of total cone cells, have resulted in a scarcity of related research. Existing studies mainly focus on investigating the underlying pathological mechanisms rather than developing viable treatments. Therefore, there is an urgent need for researchers to address and find solutions for the treatment of blue-yellow color blindness.

## 4 Summary and perspectives

Advances in various technologies have greatly enhanced our comprehension of the clinical symptoms and underlying pathological mechanisms of congenital color vision disorders. Nonetheless, there are still instances where the etiology of the disease remains inexplicable, and our grasp of the underlying cellular and molecular mechanisms is not yet complete. Further in-depth research by dedicated scientists and researchers is necessary to fill the knowledge gaps and expand our understanding of congenital color vision disorders.

Achieving cell-level resolution imaging holds paramount importance for future diagnosis and research in the field of congenital color vision disorders. Split-detection adaptive optics scanning laser ophthalmoscopy (AOSLO) imaging is a promising tool for objectively visualizing and assessing the structure of living cells. The high-resolution capabilities of split-detection AOSLO imaging enable detailed examination of cellular-level changes and abnormalities, providing valuable insights into the pathophysiology of these disorders. Its potential as a tool for diagnosis and research tool makes it a compelling avenue for further investigation within the field.

Currently, there is a shortage of effective physical treatment methods for congenital color vision disorders. Likewise, biological-level interventions, including gene therapy, are in the early stages of development and necessitate further research prior to their clinical implementation. Although gene therapy approaches are primarily being studied in animal models or clinical trials, there have been promising advancements and encouraging results have been observed in some studies, indicating potential for future treatment of these disorders in humans. The ongoing research and progress in this field offer hope for the development of effective therapies in the future.

While studies have shown therapy assisted by gene therapy can provide therapeutic effects of gene therapy in middle-aged and elderly models of color blindness, current research indicates that treatment interventions for color blindness are most effective when implemented during childhood, especially before the age of 6. It is believed that early intervention during the developmental stages of visual processing provides the best opportunity for optimal therapeutic outcomes. Although performing injection surgery on young patients presents additional technical challenges, the potential benefits of comprehensive treatment make it a worthwhile consideration.

In summary, while there is still a long journey ahead to fully understand the pathological mechanisms of color vision disorders and develop safe and effective treatments, the prospect of finding a cure is promising and warrants our unwavering commitment and perseverance.

## Author contributions

ZY: Writing – original draft. LY: Writing – original draft. WZ: Writing – original draft, Writing – review & editing. JQ: Writing – original draft, Writing – review & editing. WA: Visualization, Writing – original draft. KY: Supervision, Writing – review & editing.

## Glossary

**Table:** 

ACHM	Achromatopsia
CDS	Cone dysfunction syndromes
IRD	Inherited retinal disease
OCT	Optical coherence tomography
AOLSO	Adaptive optics scanning laser ophthalmoscopy
CD	Cone dystrophy
CRD	Cone-rod dystrophy
EZ	Ellipsoid zone
IS	Inner segment
OS	Outer segment
RPE	Retinal pigment epithelial
ER	Endoplasmic reticulum
CNG	Cyclic nucleotide gated
PDE	Phosphodiesterase
UPR	Unfolded protein response
IRE-1	Inositol requiring enzyme-1
COS	Cone outer segment
ROS	Rod outer segment
GRK-1	G protein coupled receptor kinase 1
GRK-7	G protein coupled receptor kinase 7
GC	Guanylate cyclase
GCAP	Guanylate cyclase activating protein
IPM	Interphotoreceptor matrix
LCR	Locus control region
AAV	Adeno-associated virus
rAAV	Recombinant adeno-associated virus
Gnat^cpfl3^	Cone photoreceptor function loss-3
ERG	Electroretinogram
OMR	Optomotor response
SR	Subretinal
CNTF	Ciliary neurotrophic factor
GNPs	Gold nanoparticles
CAD	Color assessment and diagnosis
RGR	RPE retinal G protein-coupled receptor
CRALBP	Cellular retinaldehyde-binding protein
OPN1MW^−/−^	M-opsin knockout
CNGA3	Cyclic nucleotide gated channel alpha 3
CNGB3	Cyclic nucleotide gated channel beta 3
GNAT2	G protein subunit alpha transducin 2
PDE6C	Phosphodiesterase 6C
PDE6H	Phosphodiesterase 6H
ATF6	Activating Transcription Factor 6
OPN1SW	Opsin 1, short wave sensitive
OPN1MW	Opsin 1, medium wave sensitive
OPN1LW	Opsin 1, long wave sensitive
